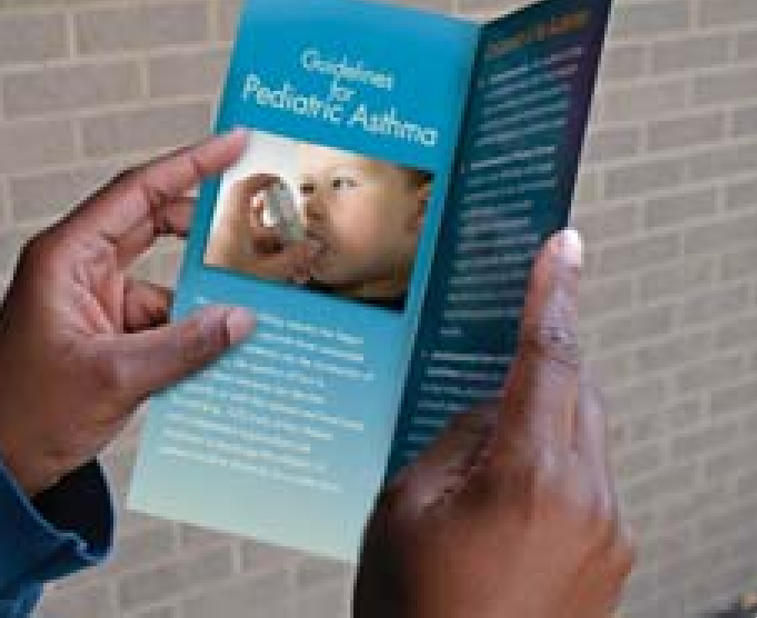# The Beat

**Published:** 2008-01

**Authors:** Erin E. Dooley

## GreenChill Partnership

Several U.S. supermarket chains have teamed up with the EPA and the refrigerant and refrigeration industries in a voluntary program to curb emissions that can exacerbate climate change. The GreenChill Advanced Refrigeration Partnership seeks to reduce refrigerant emissions over regulatory requirements as well as assess the performance of new refrigeration technologies. The 10 supermarket partners have also committed to use only ozone-friendly refrigerant alternatives and technologies in all their new and remodeled stores. Besides the environmental payoff, the EPA estimates these efforts will save the markets more than $12 million each year in increased energy efficiency and reduced operating expenses.

**Figure:**
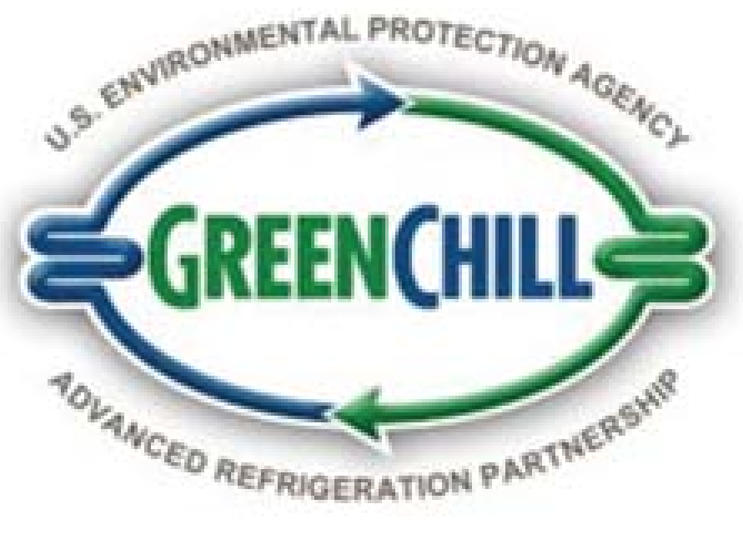


## Nonsmokers Benefit from Bans

Hundreds of municipalities across the United States have enacted some form of public smoking ban. Now a first-of-its-kind study published in Volume 37, Issue 3 (2007) of the *Journal of Drug Education* has looked at how these bans affect the health of nonsmokers. Results from 2 Indiana counties showed that hospital admissions for heart attacks among nonsmokers with no risk factors for heart disease dropped 70% within 22 months of a smoking ban going into effect. Admissions for heart attacks among smokers didn’t change, however. Secondhand smoke causes blood vessels to constrict and reduces the amount of oxygen that can be transported in the bloodstream.

## Malawi Fights Aflatoxins

Malawi is home to several efforts to help farmers rid their peanut crops of aflatoxins—waste products of *Aspergillus* molds that grow on improperly stored crops. Once a chief export, Malawian peanuts are now banned in the European Union because of the threat of aflatoxin contamination. An NGO known as the Food and Chemical Safety Environmental Action Team is educating Malawian farmers on safety measures, including better grain sorting and storage methods. The National Smallholder Farmers’ Association of Malawi also is helping farmers meet aflatoxin safety requirements for exporting their products. This collaborative encourages better planting, harvesting, and drying practices and has trained more than 800 staffers and farmers to date. In addition, the International Crop Research Institute for the Semi-Arid Tropics has set up labs in Malawi and other countries that offer affordable enzyme-linked immunosorbent assay testing for rapid sampling of crops.

**Figure:**
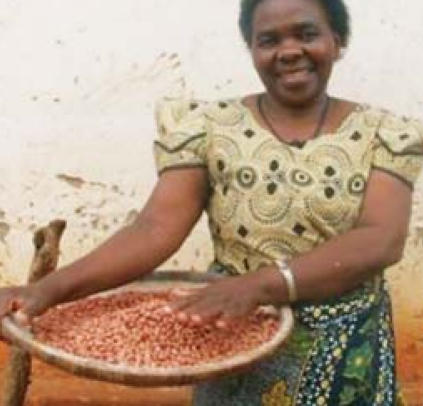


## Management Key To Asia’s Future Water Supply

One of the main messages of *Asian Water Development Outlook 2007*, a report by the Asian Development Bank, is that proper management, not volume, is the key to maintaining sufficient water supplies in Asia in the face of increasing urbanization, industrialization, population growth, and climate change. The report emphasizes that current trends in wastewater management make it harder and more expensive to provide clean water. National policies must also consider the water needs of the energy sector, an often overlooked draw on resources. The report states that Asia has the expertise and technology to ensure an adequate supply of clean water—if that expertise and technology can be fully implemented.

**Figure:**
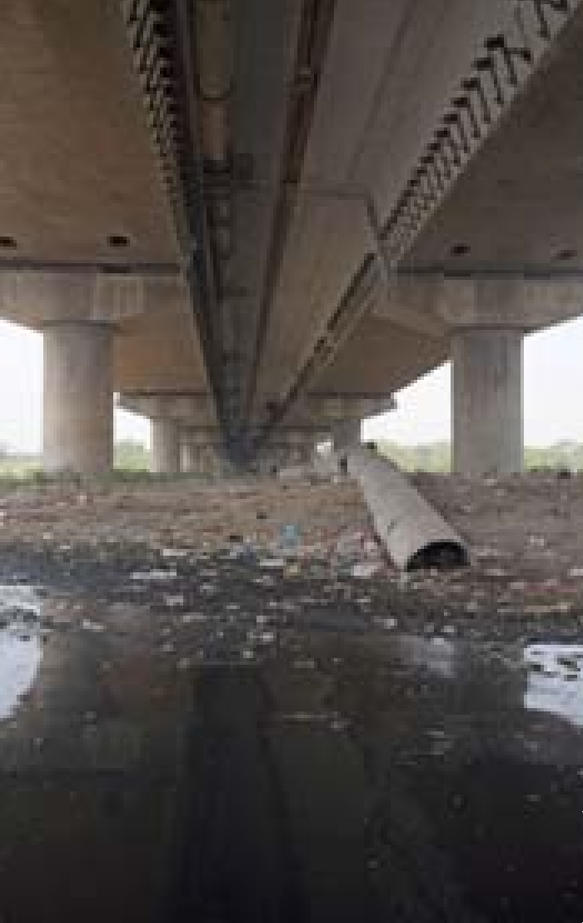


## A Helping Hand for HCFCs

A September 2007 adjustment to the Montréal Protocol called for production and consumption of hydrochlorofluorocarbons (HCFCs) to phase out in developing countries by 2013, 10 years ahead of the original Protocol schedule. Now UNEP and the Swedish EPA have announced they will help developing countries identify commercially available alternatives to achieve this goal. UNEP has also launched the HCFC Help Centre at http://www.unep.fr/ozonaction/topics/hcfc.asp to educate policy makers about HCFCs and their alternatives. Sweden has largely phased out HCFCs, which were used to replace ozone-depleting chlorofluorocarbons before they too were found to harm the ozone layer and significantly contribute to climate change.

## Asthma Brochures Fail Test

Governments need to spend health dollars in ways that can benefit the most people. A study in the Autumn 2007 issue of *Ethnicity & Disease* looked at the cultural specificity of asthma education brochures distributed in Wisconsin and found that many were ineffective at educating vulnerable populations about asthma care and prevention. The authors found several translation errors in Spanish-language materials and a lack of attention to cultural issues relevant to American Indian groups (such as the use of smudging and a tendency to distrust maintenance asthma medication). There also were no materials aimed specifically at black residents, who make up a large part of the Wisconsin population and have a disproportionately high incidence of asthma. The study used a cultural competency analysis tool developed by the Wisconsin Asthma Coalition Disparities Workgroup, which the authors say could serve as a model for other states seeking to assess their own materials.

**Figure:**